# Supplementary figures and images for: What is a “Good” figure: Scoring of biomedical data visualization
Source: PLoS One. 2025 Nov 26;20(11):e0336917. doi: 10.1371/journal.pone.0336917 (PMC12654910; doi:10.1371/journal.pone.0336917)

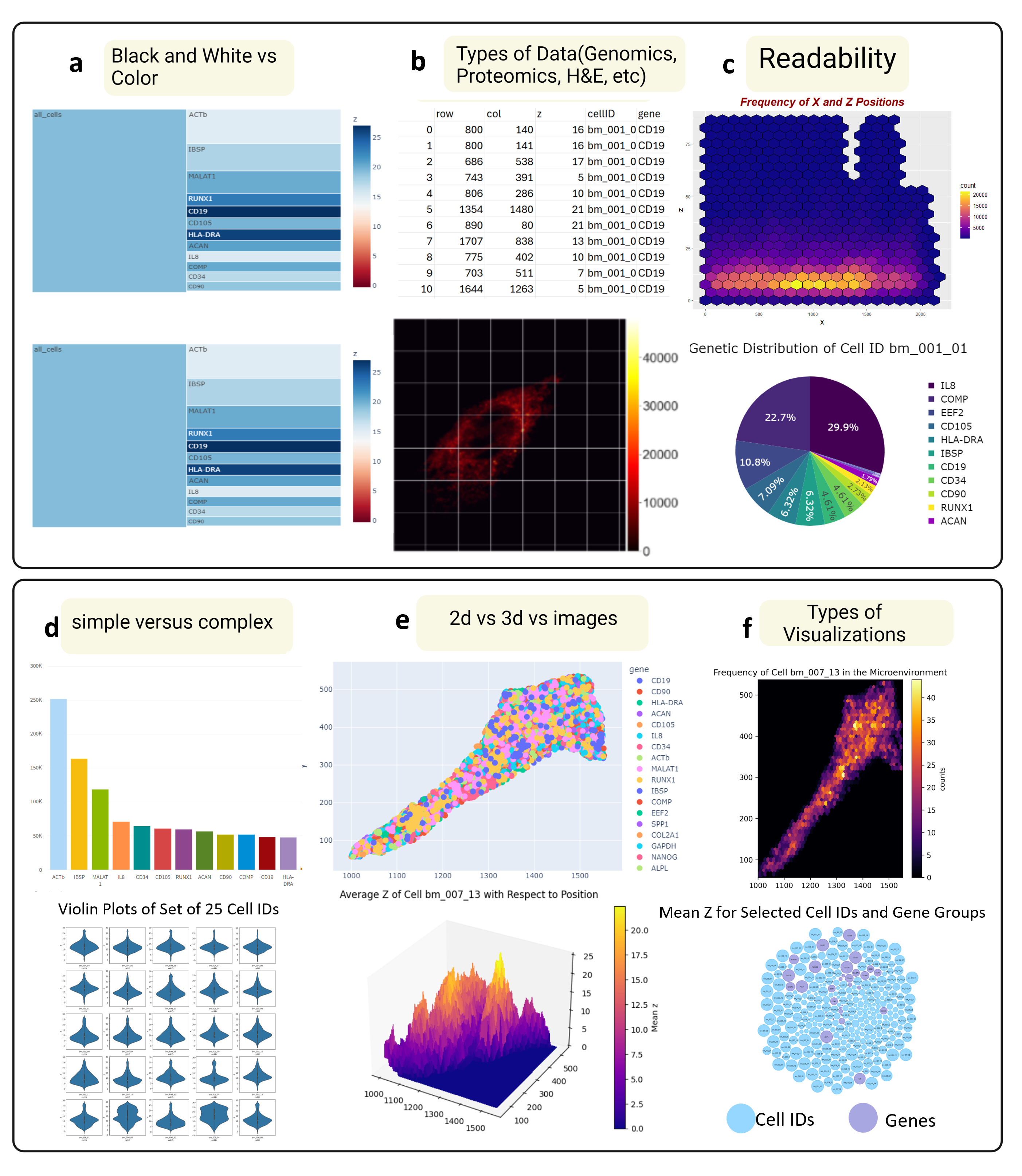

Supplement: S1 Fig — This figure illustrates different visual elements assessed by the M.E.D.V.I.S. algorithm, along with examples representing the two ends of each spectrum. (a) Color usage: the top visualization uses full color, while the bottom is presented in black and white. (b) Data type: Examples range from structured spreadsheet data to unstructured image data, illustrating the diversity of input types. (c) Readability: the top figure has low readability, especially in the axes, whereas the bottom figure demonstrates improved clarity. (d) Graph type: Two distinct visualization styles— a summative pie chart and a descriptive heat map—represent the range of graph types possible with the same data. (e) Dimensionality: This panel shows how data can be represented in different dimensions, with both 2D and 3D plots depicted. (f) Complexity: The top figure shows a simple graph with minimal visual elements, while the bottom figure displays a more complex visualization containing multiple elements. Created with BioRender.com. (TIF) [file pone.0336917.s001.tif]

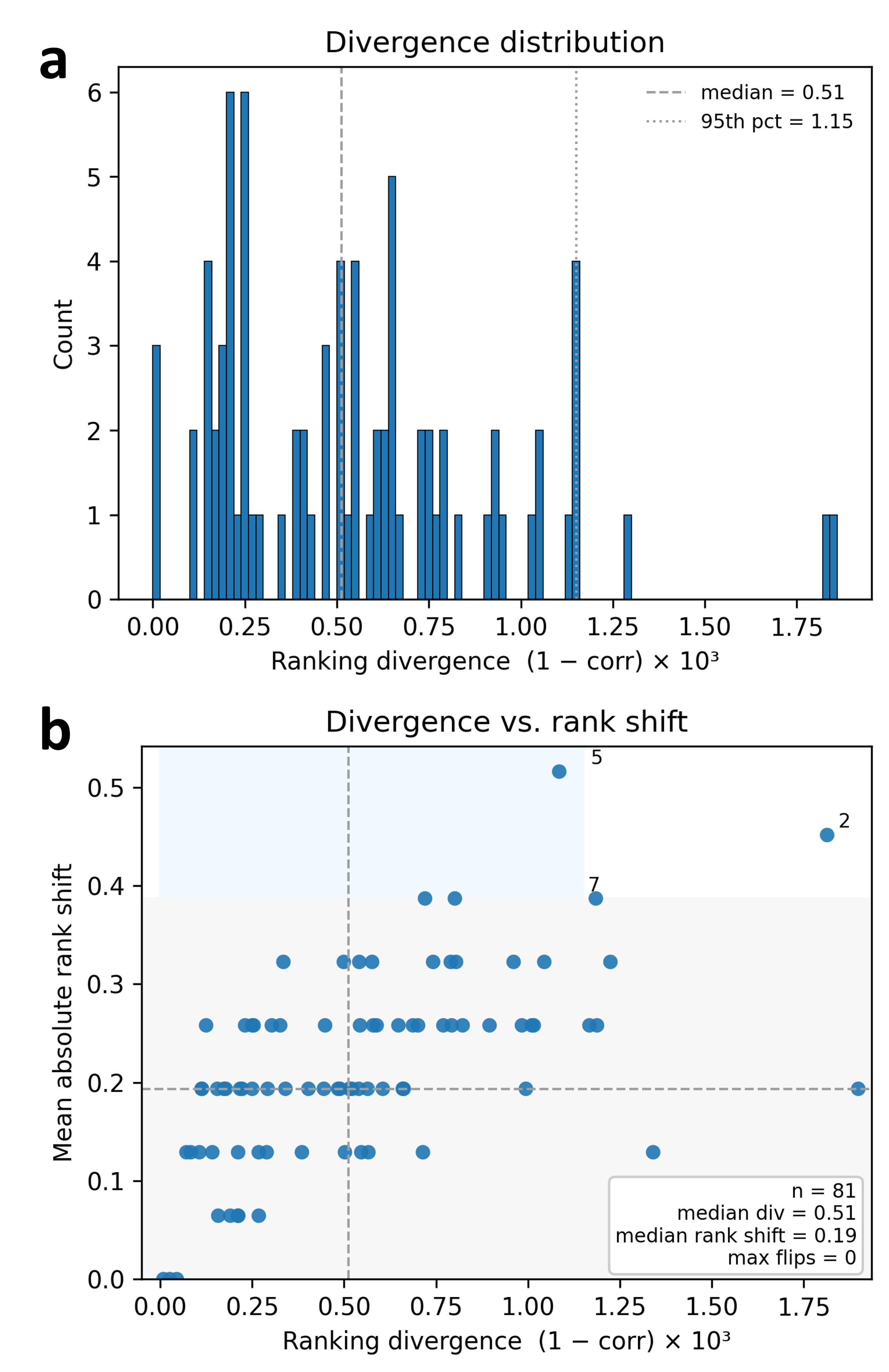

Supplement: S2 Fig — (a) Histogram of ranking divergence values (1 – corr) x 103 across all weight perturbations (n = 81). Vertical dashed and dotted lines indicate the median and 95th percentile, respectively. (b) Scatter plot of ranking divergence versus mean absolute rank shift, with bubble size encoding the number of classification flips relative to baseline. Dashed lines mark medians, and shaded regions indicate 95th-percentile stability zones. (TIF) [file pone.0336917.s002.tif]

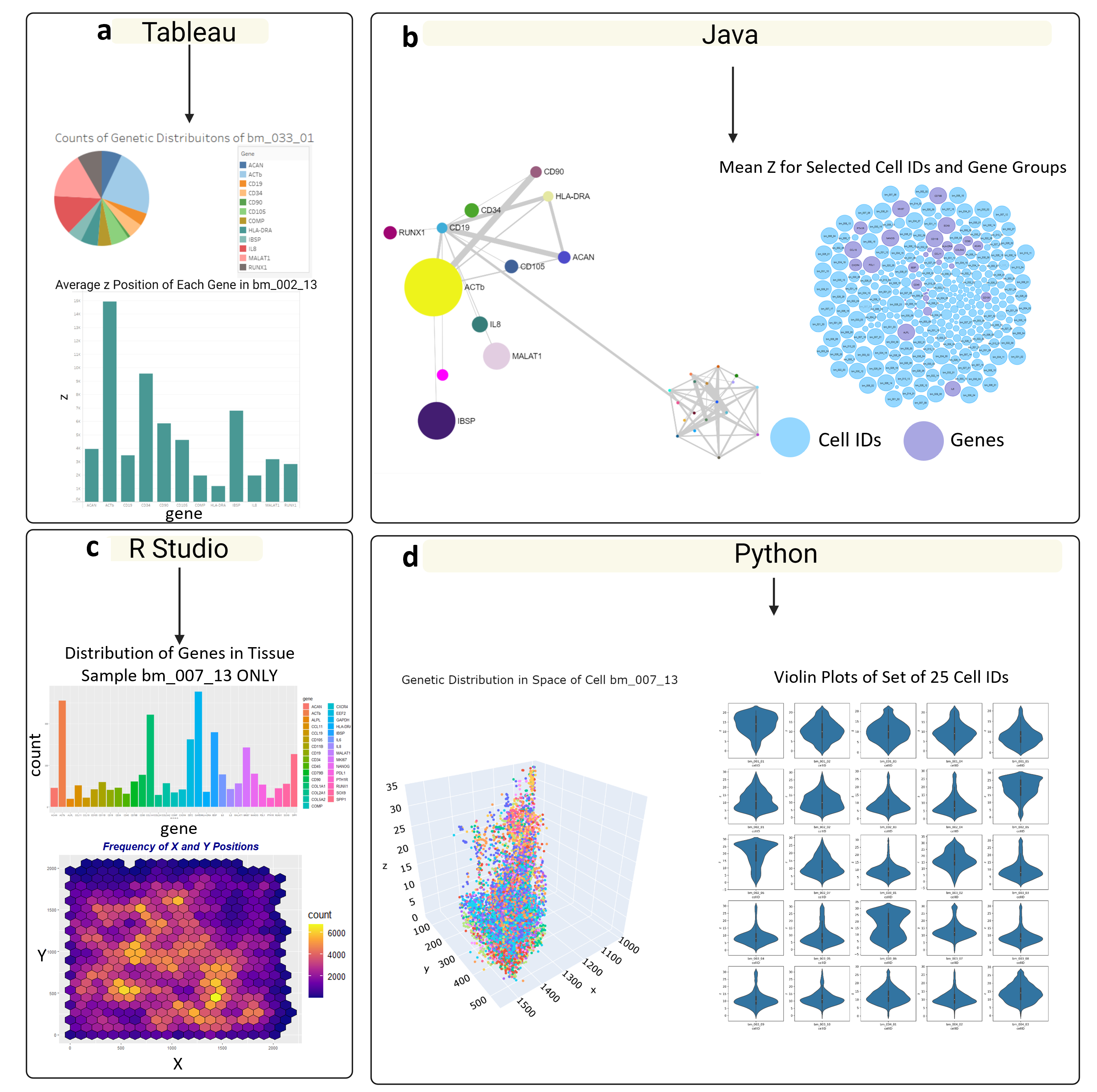

Supplement: S3 Fig — (a) A Pie Chart and a bar chart that was created using Tableau. (b) Network and Bubble Charts were created using a Java-based environment. (c) Depictions of a bar chart and a heat map that were created using R Studio. (d) Visualizations created using Plotly and Matplotlib.pyplot libraries in Python. Created with BioRender.com. (TIF) [file pone.0336917.s003.tif]

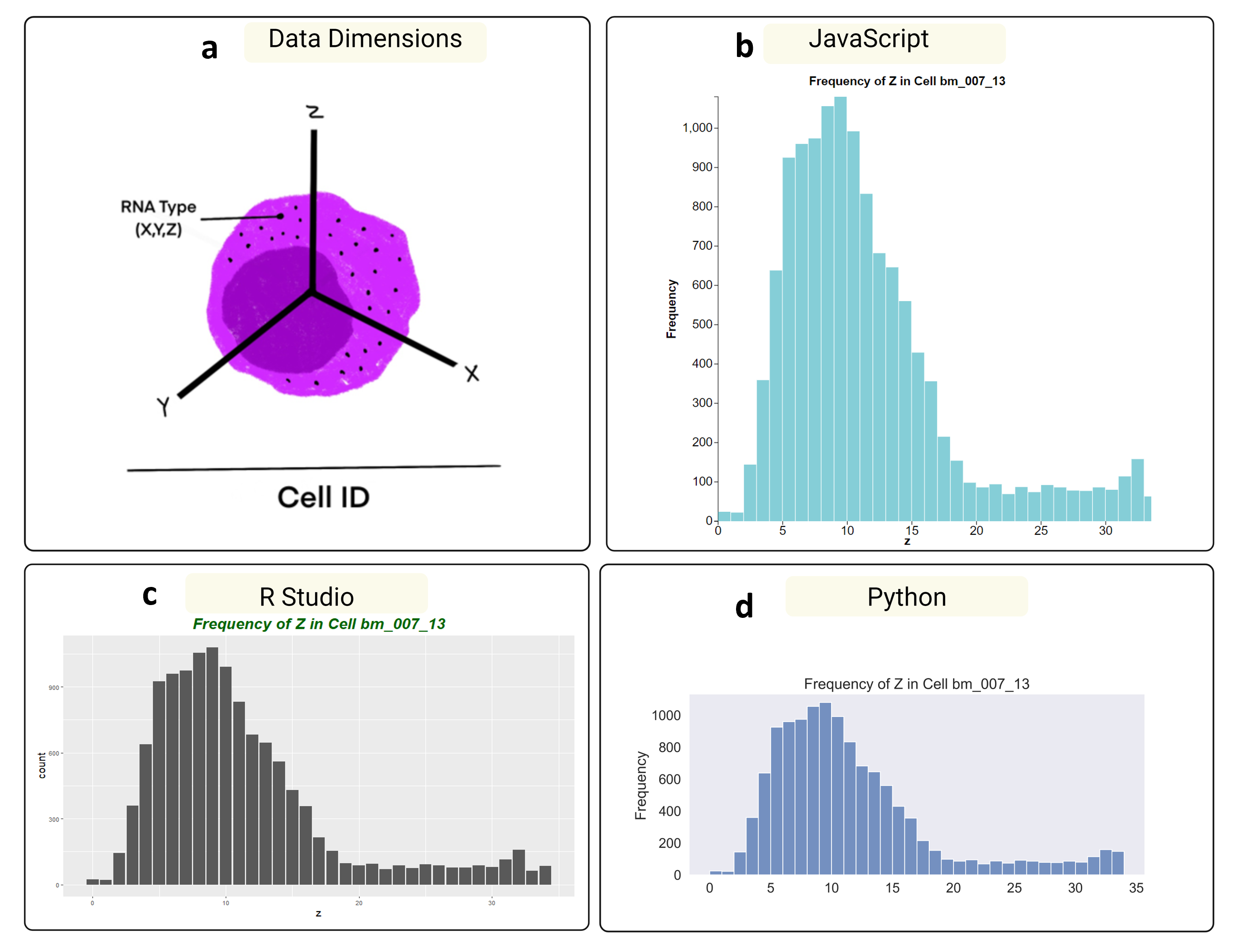

Supplement: S4 Fig — (a) Depicts the five dimensions of the data used including the X, Y, and Z positional data; the name of the mRNA at that position, and the cell ID number where it came from. (b) The Distribution of the Z positional data across all bone marrow cells produced in R. (c) The Distribution of the Z positional data across all bone marrow cells produced in Python. (d) The Distribution of the Z positional data across all bone marrow cells produced in Java. Created with BioRender.com. (TIF) [file pone.0336917.s004.tif]

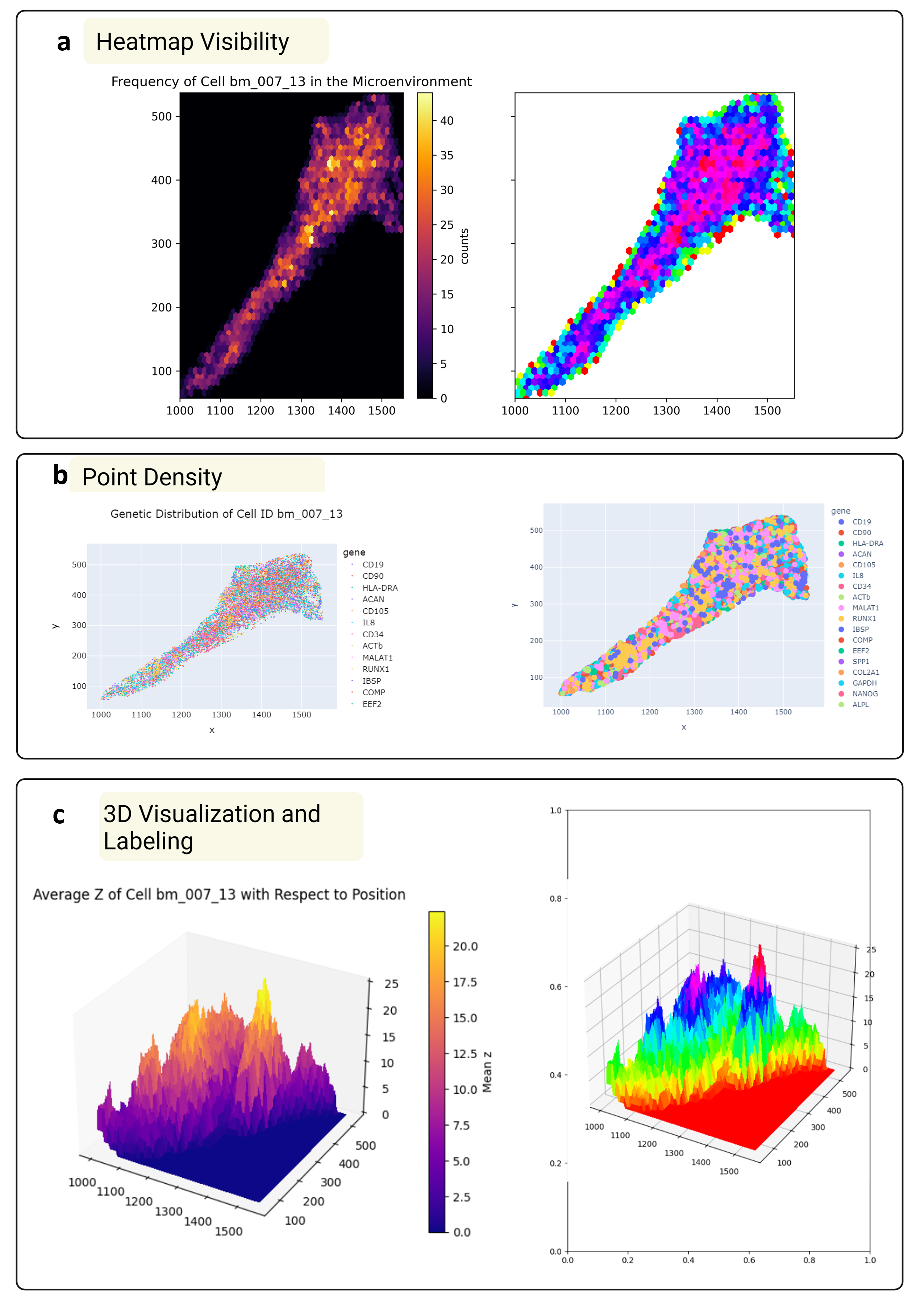

Supplement: S5 Fig — (a) A depiction of different heat maps depicting the relative frequencies of X and Y positions of various genes across the cellular microenvironment. The leftmost figure depicts easily distinguishable variations in positional frequency and depicts the scale clearly while the rightmost heatmap is difficult to distinguish different regions due to the color and does not have a scale for intensity. (b) A depiction of the overall genetic distribution of cells across X and Y positions. The topmost figure has smaller markers, a title, and appropriate text sizes while the bottommost figure has larger markers making it difficult to distinguish regions of gene markers. (c) 3D Charts that depict the x, y, and z position. The leftmost graph is good as it also depicts a fine gradient for the intensity of the z position whereas the rightmost visual is much more colorful and is difficult to easily see that same information. Created with BioRender.com. (TIF) [file pone.0336917.s005.tif]

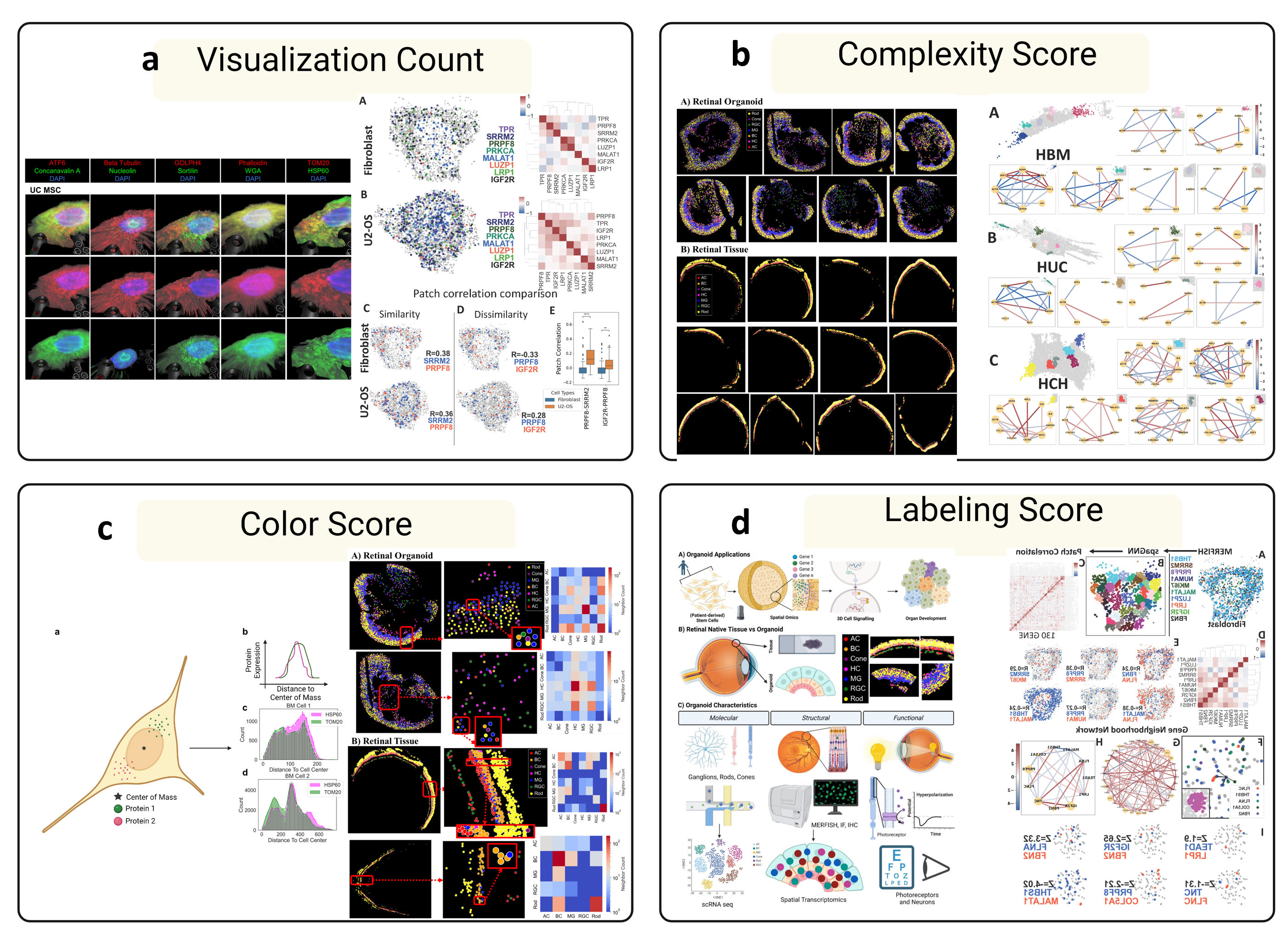

Supplement: S6 Fig — If multiple maximum or minimum were found, only the first maximum or minimum is depicted. (a) The minimum and maximum determined visualization count. (b) The minimum and maximum complexity score. (c) The minimum and maximum color score. (d) Binary representations of what is considered a “bad” and a “good” figure. Created with BioRender.com. (TIF) [file pone.0336917.s006.tif]

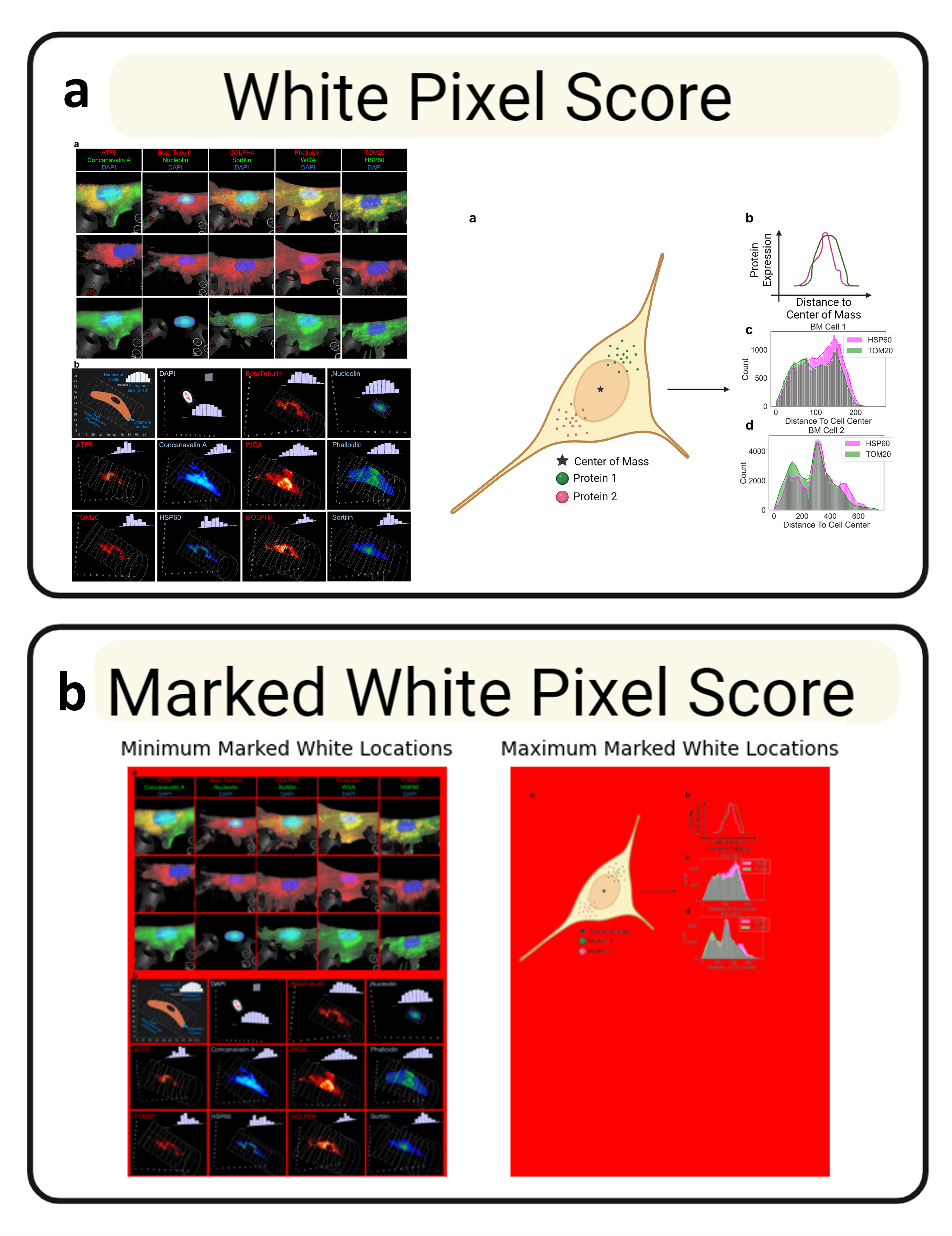

Supplement: S7 Fig — If multiple maxima or minima were found, then only the first of each was used in this visualization by the scoring algorithm. (a) The minimum and maximum determined amounts of white pixels found. (b) A Red marking of all of the white pixels that were found by the scoring algorithm. Created with BioRender.com. (TIF) [file pone.0336917.s007.tif]

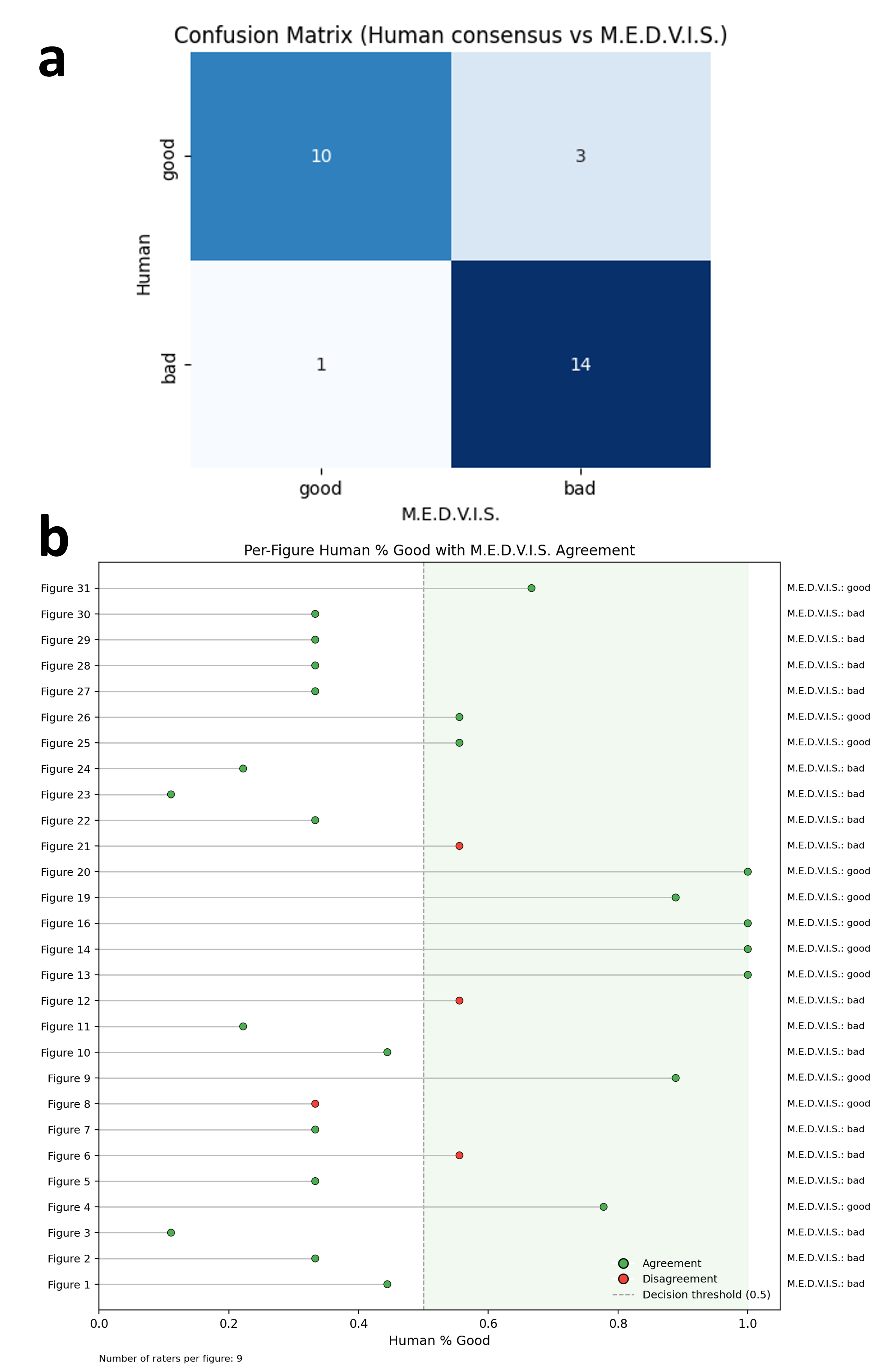

Supplement: S8 Fig — (a) Confusion matrix showing agreement between human majority votes (Good vs. Bad) and algorithm predictions. (b) Per-figure lollipop plot of the proportion of human raters voting Good (horizontal axis), with points colored green when the algorithm agreed and red when it disagreed. The shaded region indicates the decision threshold (≥0.5 = Good), and right-side annotations show the algorithm’s assigned label for each figure. (TIF) [file pone.0336917.s008.tif]
