# Supplementary material for: What is a “Good” figure: Scoring of biomedical data visualization
Source: PLoS One. 2025 Nov 26;20(11):e0336917. doi: 10.1371/journal.pone.0336917 (PMC12654910; doi:10.1371/journal.pone.0336917)
Supplement: S1 File — This manual provides detailed setup and usage instructions, including file organization, required Python libraries, output folder descriptions, and optional validation steps for automated visualization counts. (DOCX) [file pone.0336917.s009.docx]

**M.E.D.V.I.S. Instruction Manual**
*Metrics for Evaluation and Discretization of Biomedical Visuals using an Iterative Scoring Algorithm*

**Getting Started**

1. **Organize Input Files**
   - Place all figure image files in a folder titled **raws** in the same directory as the code.
   - Each image file should be named using the format **Figure x.jpg**, where x is a number (e.g., Figure 1.jpg, Figure 2.jpg, etc.).
   - The file format may vary (.jpg, .png, .tif), but the name must begin with Figure followed by a space and a number.
2. **Prepare Captions**
   - Create a folder named **captions** in the same directory.
   - For each figure, save the caption as a .txt file with the **exact same name** as the figure image (e.g., Figure 1.txt).
   - Ensure that the caption text does **not contain any special characters**. If an error occurs, inspect the file for unsupported characters and remove them.

**Required Python Libraries**

Ensure the following Python libraries are installed before running the M.E.D.V.I.S. code:

- cv2 (OpenCV)
- Pillow
- numpy
- pandas
- plotly
- matplotlib.pyplot
- pathlib
- os
- scipy
- sklearn
- umap-learn
- kaleido

Use pip install <library-name> to install any missing packages.

**Output Folder Descriptions**

The following folders will be automatically generated by the M.E.D.V.I.S. pipeline:

**1. Figure Outputs**

- Contains all generated figures, including histograms, PCA/t-SNE/UMAP plots, and maximum/minimum metric examples.

**2. Gauge Charts**

- Contains gauge plots that show how each figure scores on the four metrics: whitespace, complexity, color usage, and number of visualizations.

**3. Output Files**

- Contains final CSV files that summarize figure scoring and clustering:
  - BinaryLabels.csv: “Good” vs “Not Good” labels
  - TSNE_Labels.csv, PCA_Labels.csv, UMAP_Labels.csv: Cluster labels from each dimensionality reduction method
  - ConsensusLabels.csv: Final classification after consensus
  - FigureSuggestions.csv: Figure-specific suggestions and feedback

**4. Spreadsheets**

- Contains individual metric spreadsheets (e.g., whitespace counts, complexity scores) for all figures.

**Visual Count Validation (Code 04)**

To compare automated visualization counts to manual annotations:

1. Create an Excel file titled **testDataManualVizCount.xlsx** and save it in the root directory.
2. The Excel file must contain two columns:
   - **Figure Number**: The file name without the extension (e.g., Figure 1)
   - **Visualization Count**: The manual count for that figure
3. Sort the entries in ascending **alphanumeric order** (e.g., 1 → 10 → 11).
4. Open Code_04.py and **uncomment the chi-squared section** (by removing the triple quotes ''') to enable comparison.
5. If not validating manually, you may leave the chi-squared section commented out without any issues.

**Additional Notes**

- M.E.D.V.I.S. requires **multiple figures** for accurate clustering and scoring; it is not designed to evaluate single figures in isolation.
- The accuracy of visualization count detection depends on **proper caption formatting**, especially with labeled subpanels like “(A)” or “(b)”.
- Gauge chart outputs offer **visual diagnostics and actionable feedback**, helping users refine their figures iteratively.
- While the tool provides algorithmic suggestions, **final judgment and design choices should be made by human reviewers**.
