# Supplementary material for: What is a “Good” figure: Scoring of biomedical data visualization
Source: PLoS One. 2025 Nov 26;20(11):e0336917. doi: 10.1371/journal.pone.0336917 (PMC12654910; doi:10.1371/journal.pone.0336917)
Supplement: Attachment — Submitted filename: PLOS ONE Rebuttal Letter.docx [file pone.0336917.s011.docx]

**Rebuttal Letter**
Manuscript Title: *What is a “Good” Figure: Scoring of Biomedical Data Visualization*
Manuscript Number: PONE-D-25-05417

Dear Reviewers,

We thank the editor and reviewers for their thoughtful evaluation of our manuscript. We are grateful for the opportunity to revise and resubmit our work, and we have made extensive improvements to address each point raised. Below, we provide detailed responses to all reviewer comments. We believe that the revised manuscript now meets the expectations for clarity, structure, rigor, and presentation outlined by the journal.

**Editorial and Journal Requirements**

**1. PLOS ONE formatting and structure requirements:**
We have revised the manuscript to fully comply with PLOS ONE formatting guidelines, including the correct title page structure, file naming, and placement of figure files as separate TIFFs. Figure 1 is now explicitly cited in the main text, and Supporting Information captions have been added to the end of the manuscript.

**2. Code and data availability:**
We confirm that all code related to the M.E.D.V.I.S. algorithm and SpatioView platform has been made publicly available via GitHub, and the datasets used for case studies have been shared through Figshare, as noted in the Data Availability Statement.

**3. Author affiliations:**
Each author has been explicitly linked to their institutional affiliation(s), and any secondary or current affiliations are marked as per PLOS ONE guidance.

**Reviewer #1**

**Comment 1: The topic is very interesting and necessary because currently there are many software for data visualization but limited articles with comparisons on them.**
*Response:* We appreciate the positive assessment and fully agree with the reviewer’s view on the relevance of comparative evaluations in visualization software.

**Comment 2: The introduction is too short; authors should provide other research articles on data visualization and clarify the novelty.**
*Response:* We have significantly expanded the Introduction to include relevant literature on biomedical visualization principles [1–4], challenges [5–17], and applications in education and industry. The revised version clearly states the problem, novelty, methodology, and key findings.

**Comment 3: Figures 3 and 4 are low quality and hard to read.**
*Response:* Figures 3 and 4 have been re-rendered at 600 DPI resolution and saved as high-quality TIFF files. Labels, font sizes, and contrast have been improved for readability.

**Comment 4: Line numbers should be added.**
*Response:* Line numbers have been added to the manuscript file as requested.

**Reviewer #2**

**Comment 1: The abstract should be structured around Objective, Methodology, Findings, and Relevance.**
*Response:* We have fully rewritten the abstract to follow this structure. The revised abstract now clearly outlines the study’s motivation, the development of M.E.D.V.I.S., the tool comparison, case study findings, and broader implications for the field.

**Comment 2: The introduction lacks contextualization, a problem statement, a methodology summary, and key findings.**
*Response:* The Introduction has been substantially revised and now includes a clear background on biomedical visualization, the motivation for our scoring framework, a summary of the M.E.D.V.I.S. algorithm, tool evaluation methods, and major results from the case studies.

**Comment 3: The article reads more like a book chapter and is not structured as a scientific article.**
*Response:* We have completely reorganized the manuscript to follow the standard scientific structure: Introduction, Results (with subsections for each case study), Discussion (including challenges, implications, limitations, and future work), and Conclusion. Each section now contains clearly defined objectives and key takeaways.

**Reviewer #3**

**Comment 1: The study has compelling motivation, but the paper lacks clarity in structure.**
*Response:* The manuscript has been restructured and rewritten throughout to improve clarity. Each major component—M.E.D.V.I.S., tool comparison, and SpatioView—is now presented as an independent but connected result within the broader context of biomedical visualization needs.

**Comment 2: Figures should be aligned more closely with text for better readability.**
*Response:* We have ensured that each figure is now clearly referenced in the text near the paragraph that describes it.

**Comment 3: The introduction is too brief and does not review related literature or explain the rationale for metrics.**
*Response:* The revised Introduction includes relevant references on figure design, visualization principles, and evaluation challenges. The rationale for choosing our four scoring metrics (complexity, color, whitespace, visual count) and how they relate to existing design heuristics are now explained in the Results section.

**Additional Improvements Noted in Revision**

- A **new Discussion section** was added to synthesize challenges, interpret findings from all three case studies, and describe future directions.
- The **Conclusion** was rewritten to summarize contributions and emphasize the role of standardization in biomedical visualization.
- The **Supporting Information** section now includes clear captions and updated figure references.
- All **figure files** were removed from the manuscript text and uploaded as individual high-resolution files per journal guidelines.

We sincerely thank the reviewers for their insightful comments. Their suggestions have led to a stronger, clearer, and more rigorous manuscript. We hope that the revised version will meet the standards of PLOS ONE and be considered favorably for publication.

Sincerely,
Ahmet F. Coskun, Ph.D.
(on behalf of all co-authors)
[ahmet.coskun@bme.gatech.edu](mailto:ahmet.coskun@bme.gatech.edu)
Georgia Institute of Technology
