# Supplementary material for: What is a “Good” figure: Scoring of biomedical data visualization
Source: PLoS One. 2025 Nov 26;20(11):e0336917. doi: 10.1371/journal.pone.0336917 (PMC12654910; doi:10.1371/journal.pone.0336917)
Supplement: Attachment — Submitted filename: Rebuttal Letter.docx [file pone.0336917.s012.docx]

**Response to Reviewers – Manuscript PONE-D-25-05417R1**
*“What is a ‘Good’ Figure: Scoring of Biomedical Data Visualization”*

**Editor Comment 1:** Further quantify the rationality of scoring weights. Although four scoring metrics have been proposed, the weights of each metric for the final “good/bad” classification are not yet clear. It is recommended to add weight sensitivity analysis or expert scoring comparisons to enhance the persuasiveness of the evaluation system.

**Response:** We have added a new section describing a Weight Sensitivity Analysis Framework, in which the weights of the four scoring metrics (complexity, color usage, whitespace, and number of visualizations) were systematically varied in ±10% increments while holding the remaining metrics constant. Across all perturbations (n = 81), rankings remained highly stable (median correlation with baseline 0.9995, range 0.9981–1.0000). No classification flips were observed, and the median mean absolute rank shift was 0.19. These findings demonstrate that the scoring system is resilient to moderate variations in metric weights, thereby supporting the rationality of the weighting scheme. We have also included new figures (Supplementary Fig. S2) to visualize this stability.

**Editor Comment 2:** SpatioView visualization display effects are weak. The article provides a detailed introduction to SpatioView, but the demonstration examples are too brief. It is recommended to supplement with screenshots of higher-resolution or typical data to enhance its visual appeal and persuasiveness.

**Response:** We have expanded Case Study 3 to include high-resolution screenshots from a representative spatial transcriptomics dataset containing over 3,000 cells (Fig. 6c). These annotated examples demonstrate the core interactive features of SpatioView, including dimensionality reduction selection, hover-based cell metadata, legend placement, and zoom functions. Together, these updates provide a clearer and more compelling illustration of SpatioView’s ability to support exploratory analysis of complex multi-omics data.

**Editor Comment 3:** Include feedback from trials of M.E.D.V.I.S. or SpatioView in actual research or teaching settings to strengthen the argument for its practicality.

**Response:** We have incorporated results from a usability trial in which nine biomedical researchers and graduate students evaluated figures using M.E.D.V.I.S. Participants rated each figure as either *Good* or *Bad*, and their consensus was compared with the automated M.E.D.V.I.S. classifications. Human raters and the algorithm agreed on 85% of figures overall, with balanced performance across both categories (precision and recall ~80–90%). This analysis demonstrates that M.E.D.V.I.S. feedback is strongly aligned with human judgment of figure quality, supporting its practicality as a reliable tool for evaluating visualizations. (Supplementary Fig. S8)
